# Popliteal sciatic nerve block versus intrathecal anesthesia for Achilles tendon rupture repair surgery: a mono-centric retrospective comparative study

**DOI:** 10.3389/fmed.2025.1516874

**Published:** 2025-07-21

**Authors:** Gao Si, Liwei Wang, Minzhi Deng, Yihan Sun, Yuan Cao, Jixing Fan, Tengjiao Zhu, Yun Tian, Yang Lv, Changyi Wu

**Affiliations:** ^1^Department of Orthopedics, Peking University Third Hospital, Beijing, China; ^2^Department of Anesthesiology, Peking University Third Hospital, Beijing, China; ^3^School of Basic Medical Sciences, Peking University Health Science Center, Peking University, Beijing, China

**Keywords:** spinal anesthesia, popliteal sciatic nerve block, Achilles tendon rupture repair surgery, analgesia, postoperative complications

## Abstract

**Background:**

To compare the anesthetic and analgesic effects of popliteal sciatic nerve block and intrathecal anesthesia in acute Achilles tendon rupture patients undergoing surgery.

**Methods:**

This retrospective cohort study analyzed 115 patients with acute Achilles tendon rupture who underwent surgery at Peking University Third Hospital between May and November 2023. After excluding cases lost to follow-up or declining participation, 96 patients were ultimately enrolled. Patients were divided into two groups based on the different anesthesia methods they received: the popliteal sciatic nerve block group (BG) and the spinal anesthesia group (SAG). The anesthesia effects intraoperatively were compared between the two groups using puncture satisfaction, immediate complications of puncture, anesthesia operation time, and puncture pain evaluation. Postoperative analgesic and anesthesia recovery effects were compared between the two groups using visual analog scale (VAS), analgesic satisfaction score, sleep score, and time to complete sensory recovery. Ankle joint mobility was used to compare postoperative motor recovery between the two groups.

**Results:**

Statistical analysis revealed that in terms of anesthesia effectiveness, the BG had a shorter anesthesia operation time (1.95 ± 0.40 min) than the SAG (7.44 ± 1.90 min), and the BG (5.4%) had fewer immediate puncture complications than the SAG (25.0%). Regarding analgesic effectiveness, the BG (4.10 ± 0.09) had higher analgesic satisfaction compared to the SAG (3.14 ± 0.11), and within 48 h postoperatively, wound VAS scores in the BG were consistently lower than those in the SAG. Postoperatively, the time for complete sensory recovery in the affected lower limb in the BG (9.29 ± 0.41 h) was significantly longer than that in the SAG (6.09 ± 0.42 h).

**Conclusion:**

Compared to intrathecal anesthesia, the use of popliteal sciatic nerve block (PSNB) in Achilles tendon repair surgery resulted in shorter anesthesia operation time, fewer immediate puncture complications, higher patient satisfaction with analgesia, and longer duration of analgesic effect. PSNB may be preferred for reduced complications and prolonged analgesia.

## Highlights

• Research Question: Compare the anesthetic and analgesic effects of popliteal sciatic nerve block and intrathecal anesthesia in acute Achilles tendon rupture patients undergoing surgery.
• Findings: Compared to intrathecal anesthesia, the use of popliteal sciatic nerve block in Achilles tendon repair surgery resulted in shorter anesthesia operation time, fewer immediate puncture complications, higher patient satisfaction with analgesia, and longer duration of analgesic effect.
• Meaning: This study aims to provide clinical evidence for selecting the most suitable anesthesia method for Achilles tendon repair surgery by comparing the effects of the two anesthesia methods.

## Introduction

According to a study in 2020, it was found that there are 5–50 cases of acute Achilles tendon rupture per 100,000 individuals (1). Furthermore, with the increasing enthusiasm for physical activity among the population, the number of cases of acute Achilles tendon rupture is also increasing annually (2). Conservative treatment and surgical treatment are the two main therapeutic approaches for acute Achilles tendon rupture (3). Currently, intrathecal anesthesia is predominantly utilized in clinical practice for Achilles tendon repair surgery (4). However, some studies have suggested that the use of intrathecal anesthesia may lead to more severe complications, such as post-dural puncture headache and back pain (5), as well as potential complications including hypotension, urinary retention, and fever (6). Research has shown that peripheral nerve blocks can provide anesthesia to the surgical site while preserving lower limb motor function. This approach not only extends postoperative analgesia duration but also reduces the incidence of complications with relatively minor impact on patients’ vital signs (7). Therefore, peripheral nerve blocks can serve as a novel option for anesthesia in Achilles tendon repair surgery. Besides, we hypothesized that peripheral nerve blocks would provide superior postoperative analgesia with fewer complications compared to spinal anesthesia. However, currently, there is insufficient comparative research on the intraoperative and postoperative effects of the two anesthesia methods used in Achilles tendon repair surgery. Therefore, this study aims to provide clinical evidence for selecting the most suitable anesthesia method for Achilles tendon repair surgery by comparing the effects of the two anesthesia methods. A retrospective analysis of the intraoperative and postoperative anesthesia and analgesic effects of patients undergoing Achilles tendon repair surgery from May 2023 to November 2023 in our hospital was conducted in this study.

## Materials and methods

This was a monocentric retrospective comparative study performed at the Department of Orthopedics of Peking University Third Hospital. Ethical approval for this study (Approval No. IRB00006761-M2020252) was obtained from Peking University Third Hospital Medical Science Research Ethics Committee.

### Inclusion/exclusion criteria

The inclusion criteria were as follows:

• adult patients (≥ 18 years old);
• American Society of Anesthesiologists (ASA) physical status of I–III;
• patients undergone acute Achilles tendon rupture repair surgery by the same orthopedic surgeon;
• patients undergone popliteal sciatic nerve block (PSNB) or spinal anesthesia by the same anesthetist;
• patients whose medical records were fully accessible.

The exclusion criteria were as follows:

• patients with neuropathic disease of the affected limb;
• patients with mental disorders;
• patients with contraindications to spinal anesthesia and PSNB;
• patients where the treatment protocol could not be fully applied for different reasons.

### General information

A total of 115 patients who underwent Achilles tendon repair surgery at Peking University Third Hospital from May 2023 to November 2023 were selected. Patients for whom data was incomplete were excluded, resulting in 96 patients being included, with a follow-up rate of 83.5%.

Among the cases included in this study, all patients were male. There were 56 cases (58.3%) of left Achilles tendon rupture and 40 cases (41.7%) of right Achilles tendon rupture. The mean age of the patients was 37.21 ± 6.73 years, with a mean BMI (kg/m^2^) of 25.98 ± 10.80, and a mean surgery duration of 45.55 ± 13.69 min. All patients were operated on by the same surgeon and received anesthesia from the same anesthesiologist. Among them, there were 56 cases (58.3%) in the popliteal sciatic nerve block group (BG) and 40 cases (41.7%) in the spinal anesthesia group (SAG). With the number available, there were no significant differences in baseline characteristics between the two groups (Table 1).

**TABLE 1:** Comparison of clinical baseline characteristics between the block group (BG) and the spinal anesthesia group (SAG).

Group	Number of cases	Age	BMI	Achilles tendon rupture (left/right)	Surgery duration (min)	Rupture to calcaneus distance (cm)
Blockade group	56	37.82 ± 6.57	25.86 ± 10.80	29/27	46.50 ± 13.84	4.53 ± 1.16
SAG	40	35.75 ± 7.24	26.60 ± 1.81	30/10	43.75 ± 14.96	4.39 ± 1.17
*P-*value	–	0.10	0.31	–	0.84	0.98

The rupture to calcaneus distance is defined as the shortest linear measurement along the longitudinal axis of the Achilles tendon, from the proximal torn tendon stump to the osteotendinous junction at the posterosuperior border of the calcaneal tuberosity.

### Sample size justification

A *post hoc* power analysis was conducted for the primary outcome (intraoperative anesthesia satisfaction). Using the observed effect size (Cohen’s *d* = 19.88) and α = 0.05, the study achieved > 99.9% power with the final sample (*n* = 96). This substantially exceeds conventional power thresholds, confirming robust sensitivity for this key finding.

### Investigation methods and content

In this study, patients were categorized into the popliteal sciatic nerve BG and the SAG based on the different anesthesia methods they received during operation. After surgery, both groups received oral or intravenous non-steroidal anti-inflammatory drugs for analgesia as required. The anesthesia effects during surgery between the two groups were compared using indicators such as puncture pain score, anesthesia operation time, puncture satisfaction, immediate complications of puncture, intraoperative additional medication situation, and intraoperative anesthesia satisfaction. The postoperative analgesic and anesthesia recovery effects between the two groups were compared using indicators including postoperative wound visual analog scale (VAS) scores assessed at 8, 12, 24, and 48 h postoperatively, analgesic satisfaction score, sleep score, and time to complete sensory recovery. Ankle joint activity was used to compare postoperative movement recovery between the two groups.

### Evaluation criteria

Anesthesia operation time: the time from the needle touching the skin to the completion of anesthesia drug injection.

Puncture satisfaction, intraoperative Anesthesia satisfaction, postoperative analgesia satisfaction: patient satisfaction was assessed using a 5-point Likert scale (where 1 = *Very Dissatisfied*, 2 = *Dissatisfied*, 3 = *Neutral*, 4 = *Satisfied*, 5 = *Very Satisfied*).

Pain assessment criteria: VAS scores range from 0 to 10, where 0 indicates no pain and 10 represents the most intense pain that is intolerable.

Sleep evaluation criteria: sleep quality assessment was performed using the overall sleep quality dimension of the *Richards-Campbell Sleep Questionnaire*. Participants rated their previous night’s sleep on a 10-cm visual analog scale (0 = *worst possible sleep*, 10 = *best possible sleep*), with this singular metric serving as the primary comparator between groups based on its documented capacity to reflect integrated sleep experience.

Ankle joint activity evaluation criteria: ankle joint activity is scored from 0 to 2, where 0 indicates complete immobility of the ankle joint, 1 indicates slight mobility, and 2 indicates normal mobility.

### Anesthesia method

**FIGURE 1:**
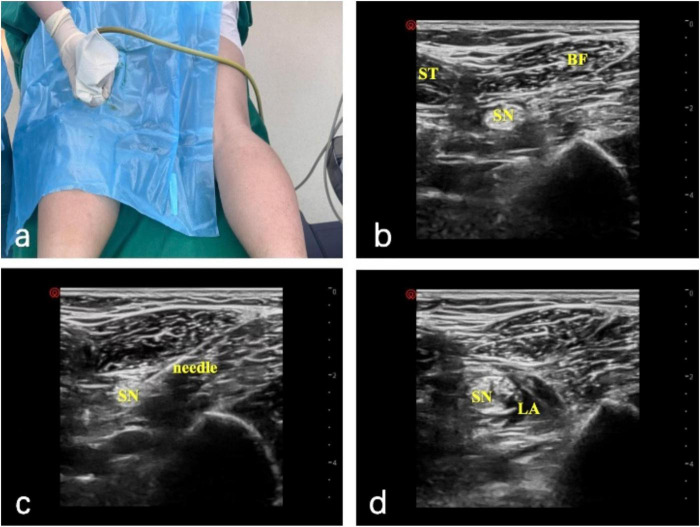
Ultrasound-guided popliteal sciatic nerve block. **(A)** Operative setup with the patient in the prone position. **(B)** Ultrasound image of the semitendinosus (ST), biceps femoris (BF), and sciatic nerve (SN). **(C)** Needle placement with a nerve stimulator (initial current 1 mA); the needle is adjusted until foot movement occurs, then repositioned to elicit plantarflexion or inversion at < 0.5 mA without aspiration of blood. **(D)** Local anesthetic (LA) distribution around the sciatic nerve (SN) after injection of 20 mL of 0.4% ropivacaine.

Popliteal sciatic nerve block: the patient was placed in the prone position, and ultrasonography was used to visualize the sciatic nerve, including the biceps femoris, semitendinosus, and semimembranosus muscles, as well as its deep aspect. 5 ml of 1% lidocaine was injected for local anesthesia. A short beveled (100 mm) 21G insulated puncture needle was connected to a nerve stimulator. The initial current was set at 1 mA, frequency at 2 Hz, and duration at 0.1 ms. The needle was inserted obliquely, and adjustments were made to its direction and depth until foot movement was observed. The needle was then tilted to induce plantarflexion or inversion of the ankle joint. When the current was reduced to less than 0.5 mA and there was still movement, and there was no blood upon aspiration, 20 ml of 0.4% ropivacaine was injected (Figure 1).

Spinal anesthesia: the L3–L4 interspace was selected as the puncture site, and a midline approach was used for puncture. The needle-through-needle technique was employed, with a 17G epidural needle first inserted into the epidural space, followed by insertion of a 25G Whitacre spinal needle into the subarachnoid space. After cerebrospinal fluid outflow, a mixture of 2 ml 0.75% bupivacaine and 1 ml 10% glucose solution was injected, totaling 3 ml, to adjust the anesthesia level to below T10.

### Surgical procedure

**FIGURE 2:**
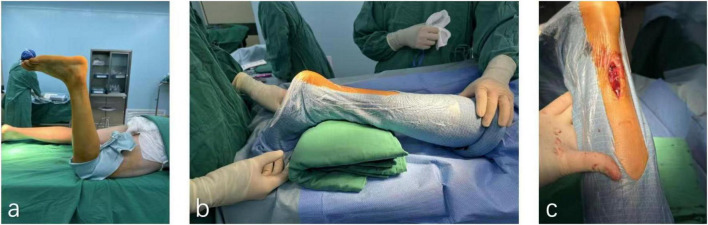
Surgical procedure for Achilles tendon rupture repair. **(a)** A patient’s leg is elevated and prepped for surgery in an operating room. **(b)** Two medical staff positions the leg, supported by a green cushion, draped in a sterile covering. **(c)** A close-up of the leg with an incision revealing underlying tissue.

Patients were positioned prone under spinal anesthesia, with a thigh-level tourniquet applied to the affected limb. Following standard iodine-alcohol disinfection and draping, a 2–3 cm midline longitudinal incision was made posterior to the ankle joint to expose the ruptured Achilles tendon. Blunt dissection of the paratenon was performed to visualize proximal and distal tendon stumps. After hematoma evacuation and irrigation, devitalized tissue was sharply debrided to optimize stump apposition. Proximal and distal tendon ends were secured with Allis clamps under axial traction to assess tension and alignment. A modified minimally invasive repair was performed using No. 2 non-absorbable polyester sutures (ETHIBOND, Johnson & Johnson, United States) in an interrupted configuration for primary tendon coaptation. To reinforce anatomical repair, a Krakow locking-loop suture technique was incorporated at the proximal stump, while the distal muscular portion of the deep tendon rupture received additional interrupted sutures for layered closure. Intraoperative tendon gliding and tension were verified by matching the repaired tendon’s resting angle to preoperative contralateral ATRA measurements. The paratenon was meticulously reapproximated with 2-0 absorbable sutures (Monocryl™, Ethicon) to minimize adhesion risks, followed by subcutaneous closure (4-0 absorbable sutures) and skin suturing. Postoperatively, a short-leg plaster cast was applied to immobilize the ankle in 15° knee-flexed equinus position (Figure 2).

All surgeries were conducted by the same team of surgeons following a standardized procedure.

### Statistical analysis

The normality of data distribution for all variables was assessed using Shapiro–Wilk tests within each group (BG and SAG). For variables satisfying the normality assumption (*p* > 0.05 in both groups), independent samples *t*-tests were used; for non-normally distributed variables, the Mann–Whitney U test was employed. To address multiple comparisons in repeated VAS measurements (4 timepoints at rest + 4 during activity), Bonferroni correction was applied within each measurement family. The significance threshold was adjusted to α = 0.0125 (0.05/4) for resting scores and separately for activity scores. Standalone outcomes (analgesia satisfaction, sleep scores, sensory recovery) maintained α = 0.05 as they constituted independent hypotheses. All analyses were performed using SPSS 27.0.

## Results

### Intraoperative anesthesia effectiveness

This study first compared the intraoperative anesthesia effectiveness between the two anesthesia methods. The puncture pain scores were 1.40 ± 0.57 in the BG and 3.15 ± 0.72 in the SAG, with a significant statistical difference. The anesthesia operation times were 1.95 ± 0.40 min in the BG and 7.44 ± 1.90 min in the SAG, also showing significant statistical difference. The puncture satisfaction scores were 4.39 ± 0.18 in the BG and 3.65 ± 0.21 in the SAG. Regarding immediate complications of puncture, in the SAG, there were 10 cases (25%) with puncture site bleeding, nerve root stimulation symptoms, hypotension, and respiratory depression during anesthesia operation, while in the BG, there were three cases (5.4%) with puncture site bleeding. All patients were clinically managed, and their symptoms were significantly relieved. In the BG, 7 cases (12.5%) used rescue analgesics, while in the SAG, 5 cases (12.5%) used vasopressors. Intergroup comparisons via Fisher’s exact tests demonstrated significant disparities in remedial analgesic use (*p* = 0.041) and vasopressor use (*p* = 0.030) between BG and SAG. The intraoperative anesthesia satisfaction scores were 4.49 ± 0.04 in the BG and 3.51 ± 0.06 in the SAG, showing significant statistical difference (Table 2).

**TABLE 2:** Comparison of puncture and intraoperative anesthesia effects between blockade group and spinal anesthesia group (SAG).

Stage	Project	Blockade group	SAG	*P*-value
Puncture procedure	Pain score for puncture	1.40 ± 0.57	3.15 ± 0.72	0.935
	Anesthesia operation time (min)	1.95 ± 0.40	7.44 ± 1.90	**0.001** *
	Puncture satisfaction	4.39 ± 0.18	3.65 ± 0.21	0.248
Intraoperative procedure	Remedy analgesic medication	7 cases (12.5%)	0 cases (0.0%)	–
	Vasoactive agent	0 cases (0.0%)	5 cases (12.5%)	–
	Intraoperative anesthesia satisfaction	4.49 ± 0.04	3.51 ± 0.06	**0.034** *

*Bolded values when *p*-value is statistically significant, with *p*-value < 0.05.

### Postoperative analgesic effectiveness

**FIGURE 3:**
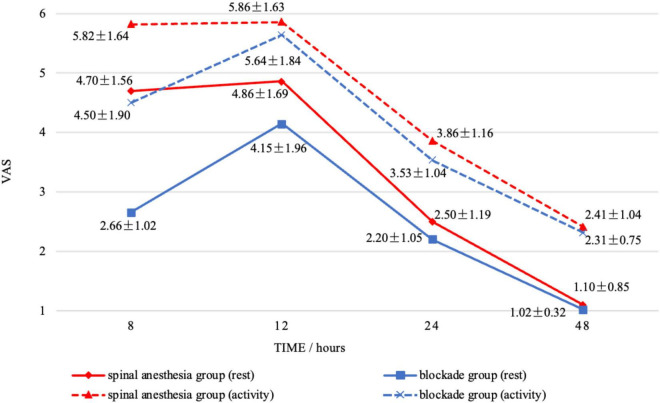
The changes in wound visual analog scale scores within 48 h postoperatively for the blockade group and the spinal anesthesia group under both rest and activity conditions were observed.

In this study, data were collected on the wound VAS scores of the two groups of patients at 8, 12, 24, and 48 h postoperatively while at rest. The wound VAS scores in the BG were 2.66 ± 1.02, 4.15 ± 1.96, 2.20 ± 1.05, 1.02 ± 0.32, respectively, while those in the SAG were 4.70 ± 1.56, 4.86 ± 1.69, 2.50 ± 1.19, 1.10 ± 0.85, respectively (Figure 3). After Bonferroni correction for four comparisons (α = 0.0125), a significant between-group difference was observed only at 8 h postoperatively (*p* < 0.0125). Throughout the first 48 h postoperatively, the wound VAS scores in the BG were consistently lower than those in the SAG. In terms of activity, the wound VAS scores in the BG were 4.50 ± 1.90, 5.64 ± 1.84, 3.53 ± 1.04, 2.31 ± 0.75, respectively, while those in the SAG were 5.82 ± 1.64, 5.86 ± 1.63, 3.86 ± 1.16, 2.41 ± 1.04, respectively (Figure 3). Following Bonferroni correction (α = 0.0125), significant differences remained only at 8 h (*p* < 0.0125). Additionally, the analgesia satisfaction in the BG (4.10 ± 0.09) was higher than that in the SAG (3.14 ± 0.11), and the sleep scores in the BG (7.31 ± 0.39) were higher than those in the SAG (7.09 ± 0.58). This study also compared the time for complete sensory recovery of the affected lower limb between the two groups, which was 9.29 ± 0.41 h in the BG and 6.09 ± 0.42 h in the SAG, with a significant statistical difference between the two groups (Table 3).

**TABLE 3:** Comparison of postoperative analgesic effectiveness and anesthesia recovery time between block group (BG) and spinal anesthesia group (SAG).

Project	Blockade group	SAG	*P*-value
Analgesia satisfaction	4.10 ± 0.09	3.14 ± 0.11	0.242
Sleep score	7.31 ± 0.39	7.09 ± 0.58	0.244
Recovery time for sensation in the affected lower limb (h)	9.29 ± 0.41	6.09 ± 0.42	**0.043** *

*Bolded values when *p*-value is statistically significant, with *p*-value < 0.05.

### Postoperative mobility recovery status

In this study, there were differences in postoperative ankle joint mobility recovery between the BG and the SAG. At 8 and 12 h postoperatively, ankle joint mobility recovery in the BG was inferior to that in the SAG. At 8 h postoperatively, in the BG, two cases (3.6%) of patients had completely immobile ankle joints, 43 cases (76.8%) had slight mobility, and 11 cases had normal mobility, while in the SAG, all patients had mobile ankle joints. At 12 h postoperatively, ankle joint mobility was present in both the BG and the SAG, but in the BG, 20 cases (35.7%) of patients had only slight mobility, and 36 cases (64.3%) had normal mobility, while in the SAG, 5 cases (12.5%) had only slight mobility, and 35 cases (87.5%) had normal mobility. At 24 h postoperatively, ankle joint mobility was normal in both the BG and the SAG (Table 4).

**TABLE 4:** Postoperative ankle joint mobility recovery status within 24 h in block group (BG) and spinal anesthesia group (SAG).

Group	Time	Ankle joint range of motion		
		0 score	1 score	2 score
SAG	8 h	0 cases (0%)	15 cases (37.5%)	25 cases (62.5%)
	12 h	0 cases (0%)	5 cases (12.5%)	35 cases (87.5%)
	24 h	0 cases (0%)	0 cases (0%)	40 cases (100%)
Blockade group	8 h	2 cases (3.6%)	43 cases (76.8%)	11 cases (19.6%)
	12 h	0 cases (0%)	20 cases (35.7%)	36 cases (64.3%)
	24 h	0 cases (35.7%)	0 cases (0%)	56 cases (100%)

## Discussion

Both spinal anesthesia and popliteal sciatic nerve blockade are commonly used anesthesia techniques in clinical practice. In recent years, with the development of ultrasound technology, peripheral nerve blockade has become more widely used. Sort et al. found that compared to spinal anesthesia, peripheral nerve blockade provided better postoperative analgesia in acute ankle fracture surgery (8). Similarly, similar conclusions could be drawn in surgeries for total knee arthroplasty (9). Therefore, this study compares the anesthesia and analgesic effects of popliteal sciatic nerve blockade and spinal anesthesia in patients undergoing surgery for acute Achilles tendon rupture, providing clinical evidence for anesthesia selection in Achilles tendon repair surgeries.

### Intraoperative anesthesia effectiveness

In this study, under the operation of the same skilled anesthesiologist, there was a significant statistical difference in the anesthesia operation time between the BG and the SAG (Table 2). During popliteal sciatic nerve blockade, ultrasound guidance allows for more precise and expedited injection localization (10). The popliteal sciatic nerve is located relatively superficially, and once the needle penetrates the skin and traverses the biceps femoris muscle, it can directly reach the surface of the sciatic nerve, facilitating relatively easy positioning (11). Additionally, the anatomical structure of the popliteal sciatic nerve is relatively consistent and is not significantly influenced by individual factors such as age (12). The puncture effectiveness of spinal anesthesia is significantly influenced by the positioning of the patient, leading to higher difficulty in puncture (13). In summary, under the same conditions, compared to spinal anesthesia, popliteal sciatic nerve blockade requires shorter procedural times.

**FIGURE 4:**
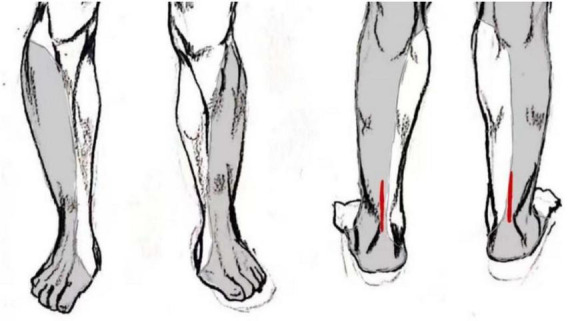
Schematic diagram of sciatic nerve sensory distribution area and locations of open surgical incisions, which in some cases extended beyond the area reliably covered by the popliteal sciatic block.

The immediate complications during puncture differ between the two anesthesia methods. In this study, the SAG experienced complications such as bleeding at the puncture site, symptoms of nerve root stimulation, hypotension, and respiratory depression in 10 cases (25%) during the anesthesia procedure. Spinal anesthesia can lead to partial sympathetic blockade, and if the blockade level is too high, it may cause relative vagal nerve dominance, further resulting in hypotension and bradycardia (14). However, with clinical interventions, patients’ symptoms can be significantly alleviated (15). In this study, there were five cases (12.5%) of spinal anesthesia patients who used vasopressor drugs intraoperatively to counteract their hypotension. In addition, two cases (5.0%) of patients were managed with oxygen supplementation via a face mask intraoperatively to counteract respiratory depression, possibly attributed to an elevated level of anesthetic blockade leading to extensive thoracic spinal nerve blockade, resulting in intercostal muscle paralysis, causing weakened thoracic breathing and heightened abdominal breathing, leading to respiratory depression (16). In this study, there were only three cases (5.4%) of puncture site bleeding observed in the BG during anesthesia procedures, and no iatrogenic nerve injuries occurred. This was attributed to the more precise anesthesia procedures facilitated by ultrasound guidance, thereby reducing the incidence of nerve damage (17). Additionally, in this study, a total of seven cases (12.5%) in the BG required intraoperative rescue analgesic medications, indicating a potential occurrence of incomplete blockade. In some cases, due to larger or more proximal incisions, the block area could not be fully covered, potentially leading to incisional pain during surgery (Figure 4). In such instances, administering additional analgesic medication resulted in satisfactory outcomes. Therefore, although PSNB predominantly affects the area innervated by the sciatic nerve, resulting in minimal systemic impact and fewer complications, there remains the possibility of requiring additional analgesic medications due to inadequate block coverage.

In terms of anesthesia satisfaction, the BG was significantly higher than the SAG. Patients undergoing PSNB experienced only sensory and motor loss in the affected knee region post-anesthesia, preserving knee joint mobility. Sensation and motor function in the unaffected lower limb were unaffected, reducing patient anxiety. Complications of intravertebral anesthesia include hypotension, respiratory depression, and urinary retention, which can impact patient satisfaction with anesthesia. Additionally, intravertebral anesthesia requires patients to assume specific positions, and post-anesthesia, there is complete loss of sensation and motor function in both lower limbs, which may cause discomfort (18). In contrast, patients undergoing PSNB exhibit higher satisfaction with anesthesia.

### Postoperative analgesic efficacy

In this study, the postoperative analgesic effect in the BG was superior to that in the SAG. A wound VAS score ≥ 4 was considered as moderate to severe pain perceived by the patients (19). At 8 h postoperatively, patients in the BG did not report significant pain sensation, whereas those in the SAG could perceive significant pain. Notably, although the active pain VAS score at 8 h postoperatively was significantly lower in the BG group compared to the SAG group (mean difference: 1.32 points), the clinical interpretation of this difference necessitates consideration of the Minimal Clinically Important Difference (MCID). Based on existing literature (20), the estimated MCID for postoperative pain VAS approximates 1.77–5.21 points. The observed difference in this study approaches this threshold. Consequently, while the BG regimen may offer a potential advantage in early postoperative active pain control, its definitive clinical significance requires further evaluation. In contrast, the BG group demonstrated a clinically meaningful reduction in resting VAS scores at 8 h postoperatively, with a mean difference of 2.04 points lower than the SAG group. Within the initial 12 h postoperatively, both groups of patients experienced noticeable pain sensations regardless of rest or activity. At 24 and 48 h postoperatively, both groups of patients reported no significant pain, yet VAS scores in the BG were consistently lower than those in the SAG (Figure 3). Although our results demonstrated lower postoperative VAS pain scores in the BG compared to the SAG, it is crucial to consider the potential confounding influence of postoperative pain management protocols. To mitigate this confounder, a standardized postoperative analgesic protocol was strictly followed for all patients: tramadol hydrochloride sustained-release tablets were administered orally twice daily as routine analgesia. Intramuscular tramadol hydrochloride injection was given as a rescue analgesic if the VAS ≥ 4.

Additionally, patients in the BG exhibited significantly longer time for complete recovery of sensation in the affected lower limb compared to the SAG, reflecting the prolonged duration of analgesia with popliteal sciatic nerve blockade. Different local anesthetics have varying rates of metabolism and half-lives (21). During popliteal sciatic nerve blockade, the local anesthetic is injected around the nerve, resulting in slower metabolism and prolonged block duration. In contrast, spinal anesthesia involves injecting the local anesthetic into the subarachnoid space, where cerebrospinal fluid metabolism is faster (22). Therefore, compared to spinal anesthesia, popliteal sciatic nerve blockade provides better postoperative analgesia due to its longer duration of action.

### Postoperative movement recovery

The study found that postoperative motor recovery time was slower in the BG compared to the SAG. Additionally, the percentage of patients with complete recovery of ankle joint activity was lower in the BG (19.6%, 64.3%) than in the SAG (62.5%, 87.5%) at 8 h versus 12 h postoperatively. However, both groups achieved complete recovery of ankle joint activity at 24 h postoperatively. Notably, BG’s slower motor recovery is not a flaw but an expected outcome. Local anesthetics used in nerve blocks usually have a longer motor-block duration compared to spinal anesthetics. Besides, popliteal sciatic nerve blockade directly inhibits motor fibers to the muscles, delaying ankle plantarflexion/dorsiflexion recovery. But, spinal anesthesia’s motor blockade is more generalized and transient.

Following Achilles tendon rupture repair surgery, routine immobilization with plaster casting is typically employed to avoid early joint movement. Plaster casting serves to stabilize and protect the freshly repaired Achilles tendon, limiting the range of motion and reducing tension, thereby facilitating tendon healing and rehabilitation (23). BG’s prolonged motor block may reduce accidental weight-bearing or active movement, protecting the repair. Besides, patients may inadvertently stress the tendon if pain is inadequately controlled, leading to reinjury or pain-induced compensatory movements. Thus, BG’s analgesic superiority outweighs transient motor delays. Consequently, maintaining a state of rest within the initial 24 h postoperatively is conducive to subsequent recovery.

### The role of adjuvants in popliteal sciatic nerve block

Some researches found that the addition of adjuvants to the local anesthetic solution in ultrasound-guided PSNB can prolong the duration of analgesia (24). For instance, dexamethasone may extend analgesia by suppressing the inflammatory response and reducing the release of inflammatory mediators in local nerve tissue (25). Furthermore, the use of adjuvants may enhance the block success rate, improve the quality of anesthesia, and increase patient comfort (26).

However, the incorporation of adjuvants in PSNB carries potential risks. It may increase the neurotoxicity of local anesthetics (27). Besides, while vasoconstriction induced by some adjuvants might prolong the local anesthetic’s effect by reducing systemic absorption, it could simultaneously compromise neural blood flow (28). In scenarios demanding optimal blood supply to promote healing, such as Achilles tendon repair, we avoid using such adjuvants. In the BG group, the surgical duration was 46.50 ± 13.84 min. The duration of analgesia provided by the local anesthetic alone was sufficient to cover the surgical procedure. Given this context, the risks associated with adjuvant use are not justified solely for the purpose of extending block duration.

### Intrathecal anesthesia and ultrasound guidance

Research indicates that ultrasound-assisted spinal anesthesia provides enhanced visualization of vertebral structures and neural elements. This facilitates the procedure by reducing the number of spinal puncture attempts and lowering the associated risk of complications (29). However, maintaining strict aseptic technique during real-time ultrasound guidance poses significant challenges. Furthermore, the adoption of this technology entails higher costs compared to conventional landmark-based techniques. Additionally, proficiency in ultrasound-assisted spinal anesthesia requires operators to possess advanced ultrasonographic skills and detailed knowledge of neuroanatomy, resulting in a steeper learning curve. For the majority of patients undergoing Achilles tendon repair, conventional landmark-based spinal anesthesia is generally sufficient. Nevertheless, in patients presenting with technical challenges such as obesity or aberrant spinal anatomy, ultrasound-guided spinal anesthesia is the preferred approach to significantly improve the likelihood of successful puncture and anesthesia delivery (30).

### Limitations and future directions

This study has several limitations that warrant consideration. The primary limitation stems from its retrospective design, which inherently introduces susceptibility to selection bias and constrains data collection standardization. Although we employed validated assessment scales, subjective measures like VAS scores remain susceptible to patient reporting bias – a concern mitigated by our use of sleep quality assessment as an objective supplemental indicator. Second, the single-center design and involvement of a single surgical team may affect the generalizability of findings to different healthcare settings. Third, the exclusive inclusion of male patients (despite the 20:1 male predominance in sports-related Achilles tendon rupture) precludes analysis of potential sex-based differences in pain perception and anesthesia responses.

To address these limitations, we propose a multicenter randomized controlled trial comparing: (1) Sex-specific outcomes using stratified recruitment (minimum 20% female participants); (2) Cost-effectiveness analyses across different surgical settings; (3) Objective pain biomarkers (e.g., pupillometry, stress hormones) to complement subjective scales; (4) Long-term functional outcomes using dynamometric measurements of ankle mobility.

## Conclusion

Compared to intrathecal anesthesia, the use of PSNB in Achilles tendon repair surgery resulted in shorter anesthesia operation time, fewer immediate puncture complications, higher patient satisfaction with analgesia, and longer duration of analgesic effect. PSNB may be preferred for reduced complications and prolonged analgesia.

## Data Availability

The raw data supporting the conclusions of this article will be made available by the authors, without undue reservation.
